# A Feature Selection Approach Based on Interclass and Intraclass Relative Contributions of Terms

**DOI:** 10.1155/2016/1715780

**Published:** 2016-08-08

**Authors:** Hongfang Zhou, Jie Guo, Yinghui Wang, Minghua Zhao

**Affiliations:** School of Computer Science and Engineering, Xi'an University of Technology, Xi'an, Shaanxi 710048, China

## Abstract

Feature selection plays a critical role in text categorization. During feature selecting, high-frequency terms and the interclass and intraclass relative contributions of terms all have significant effects on classification results. So we put forward a feature selection approach, IIRCT, based on interclass and intraclass relative contributions of terms in the paper. In our proposed algorithm, three critical factors, which are term frequency and the interclass relative contribution and the intraclass relative contribution of terms, are all considered synthetically. Finally, experiments are made with the help of kNN classifier. And the corresponding results on 20 NewsGroup and SougouCS corpora show that IIRCT algorithm achieves better performance than DF, *t*-Test, and CMFS algorithms.

## 1. Introduction

As the number of digital documents available on the Internet has been growing significantly in recent years, it is impossible to manipulate manually such enormous information [1]. More and more methods based on statistical theory and machine learning have been proposed, and they are applied successfully to information processing. An effective method for managing the vast amount of data is text categorization, which has been widely applied to many fields such as theme detection, spam filtering, identity recognition, web page classification, and semantic parsing.

The goal of text classification is to assign a new document automatically to a predefined category [2]. A typical text classification framework consists of preprocessing, document representation, feature selection, feature weighting, and classification stages [3]. In the preprocessing stage, it usually contains such tasks as tokenization, stop-word removal, lowercase conversion, and stemming. In the document representation stage, it generally utilizes the vector space model that makes use of the bag-of-words approach [4]. In the feature selection stage, it usually employs the filter methods such as document frequency (DF) [5], mutual information (MI) [6], information gain (IG) [7], and chi-square (CHI) [8]. In the feature weighting stage, it usually uses TF-IDF to calculate the weights of the selected features in each document. And in the classification stage, it always uses some popular classification algorithms, for example, decision trees [9], *k*-Nearest Neighbors (kNN) [10], and support vector machine (SVM) [11].

The major characteristic of text categorization is that the feature number in the feature space can easily reach up to tens or hundreds of thousands. It can not only increase computational time but also degrade classification accuracy [12]. As a consequence, feature selection plays a critical role in text classification.

The existing experimental results show that IG is one of the most effective feature selection methods, the performance of DF is similar to IG, and MI is the worst [13]. Through comparative analysis, it is easy to find that the performances of DF and IG are good, which means that high-frequency terms are really essential to text classification, while the performance of MI is bad as it is inclined to select low-frequency terms as features. Besides, *t*-Test method is also based on term frequency [14] and its performance is good. During feature selecting, Categorical Term Descriptor (CTD) method considers the document frequency of IDF and the category information of ICF particularly [15]. Similarly, Strong Class Information Words (SCIW) method selects the terms which have good abilities to distinguish categories [16] and it also considers the category information. Experimental results show that CTD and SCIW both have good accuracies. So we can easily know that feature selection methods based on category information always have good performances. As a result, we draw that high-frequency terms and category information are very important in improving the classification effectiveness. Comprehensively Measure Feature Selection (CMFS) method [1] considers high-frequency terms and category information simultaneously, and it also obtains good results. But it does not consider the interactions between categories. In view of these, we propose a new feature selection algorithm named as feature selection approach based on interclass and intraclass relative contributions of terms (IIRCT), in which term frequency and the interclass relative contribution and the intraclass relative contribution of terms are all considered synthetically.

## 2. Related Works

To deal with massive documents corpora, many feature selection approaches have been proposed. And their purpose is to select the terms whose classification capabilities are stronger comparatively in feature space. After feature selection, the dimensionality of feature space can be reduced, and the efficiency and accuracy of classifiers can be improved. Its main idea is as follows. Firstly, it uses the feature selection function to compute some important indicators of each word in feature space. And then, it sorts the words in descending order according to above values. Finally, it selects the top m words to construct the feature vector.

In this section, we introduce some symbols used in the following firstly.

tf_*ij*_ is the times that the term *t*_*i*_ appears in document *d*_*j*_, namely, term frequency.

$\bar{tf_{ki}}$ is the average frequency of the term *t*_*i*_ within a single category *C*_*k*_, and the calculation formula is as follows:

(1) $$\begin{array}{c} \bar{tf_{ki}}=\overset{N}{\underset{j=1}{\sum }}tf_{ij}\cdot \frac{I\left(d_{j},C_{k}\right)}{N_{k}}, \end{array}$$

where *N* is the document number in collection *D*, *N*_*k*_ is the document number in category *C*_*k*_, and *I*(*d*_*j*_, *C*_*k*_) = {1,  *d*_*j*_ ∈ *C*_*k*_; 0,  *d*_*j*_ ∉ *C*_*k*_}, which is an indicator to discriminate whether document *d*_*j*_ belongs to category *C*_*k*_.

$\bar{tf_{i}}$ is the average term frequency of the term *t*_*i*_ in collection *D*, and it is calculated according to

(2) $$\begin{array}{c} \bar{tf_{i}}=\frac{1}{N}\overset{N}{\underset{j=1}{\sum }}tf_{ij}. \end{array}$$

Similarly, *N* is the document number in collection *D*.

Then we give the definition of three feature selection methods, which are DF, *t*-Test, and CMFS, respectively.

### 2.1. DF

DF method calculates the number of documents which contain the terms in the category to measure the relevance of the terms and the categories. And the terms can be reserved only when they appear in adequate documents. This measurement is based on such an assumption that the terms which have low values of DF have few effects on the classification performance [8]. So DF method always selects terms with high values of DF and removes terms with low values of DF.

DF method is a simple word reduction technology and has good performance. Due to its linear complexity, it can be easily scaled to be used in large-scale corpus.

### 2.2. *t*-Test

*t*-Test [14] is a feature selection approach based on term frequency, which is used to measure the diversity of the distributions of a term between the specific category and the entire corpus. And it is defined as follows:

(3) $$\begin{array}{c} t\text{-}Test\left(\;t_{i},C_{k}\;\right)=\frac{\left|\bar{tf_{ki}}-\bar{tf_{i}}\right|}{\sqrt{1/N_{k}-1/N}*s_{i}}. \end{array}$$

In (3), $\bar{tf_{ki}}$ is the average frequency of the term *t*_*i*_ within a single category *C*_*k*_, $\bar{tf_{i}}$ is the average term frequency of the term *t*_*i*_ in collection *D*, *N*_*k*_ is the document number in category *C*_*k*_, *N* is the document number in collection *D*, $s_{i}^{2}=\left(1/\left(N-\left|C\right|\right)\right)\sum_{k=1}^{\left|C\right|}\sum_{j\in C_{k}}^{}(tf_{ij}-\bar{tf_{ki}}{)}^{2}$, and |*C*| is the category number in collection *D*.

The following two ways are used alternatively when the main features are finally selected:

(4) t-Testavgti=∑k=1CpCk∗t-Testti,Ck,

(5) t-Testmaxti=maxk=1C⁡t-Testti,Ck,

where *p*(*C*_*k*_) = *N*_*k*_/*N*, *N*_*k*_ is the document number in category *C*_*k*_, and *N* is the document number in collection *D*.

Generally, the method shown in (4) is always better than that shown in (5) for multiclass problem.

### 2.3. CMFS

When selecting features, DF method only computes the document frequency of each unique term in one category, and then the highest document frequency of a term in various categories is retained as the term's score. DIA association factor method [17] only calculates the distribution probability of a term in various categories, and then the highest probability of the term can be used as the term's score. Yang et al. [1] noticed that both DF and DIA methods only focus on one respect of the problems (row or column). Thus DF method concentrates on the column of the term-to-category matrix, while DIA focuses on the row of the term-to-category matrix. Based on such observation, a new feature selection algorithm, Comprehensively Measure Feature Selection (CMFS), is proposed by Yang et al. It comprehensively measures the significance of a term both in intercategory and intracategory. And it is defined as follows:

(6) $$\begin{array}{c} CMFS\left(\;t_{i},C_{k}\;\right)=p\left(\;t_{i}\;|\;C_{k}\;\right)*p\left(\;C_{k}\;|\;t_{i}\;\right). \end{array}$$

Here, *p*(*t*_*i*_∣*C*_*k*_) is the probability that the feature *t*_*i*_ appears in category *C*_*k*_, and *p*(*C*_*k*_∣*t*_*i*_) can be considered as the conditional probability that the feature *t*_*i*_ belongs to category *C*_*k*_ when the feature *t*_*i*_ occurs.

To measure the goodness of a term globally, two alternate ways can be used to combine the category-specific scores of a term. And the formulae are as follows:

(7) CMFSavgti=∑k=1CpCk∗CMFSti,Ck,CMFSmaxti=maxk=1C⁡CMFSti,Ck,

where *p*(*C*_*k*_) = *N*_*k*_/*N*, *N*_*k*_ is the document number in category *C*_*k*_, and *N* is the document number in collection *D*.

## 3. IIRCT

In this section, we propose a feature selection approach based on interclass and intraclass relative contributions of terms. In the proposed algorithm, three critical factors, which are term frequency and the interclass relative contribution and the intraclass relative contribution of terms, are all considered synthetically.

### 3.1. Motivation

At present, a large number of feature selection algorithms emerge. Through studying and analysing them, we can easily find that DF, IG, and *t*-Test algorithms are inclined to select high-frequency terms as main features, and their performances are good. Among them, DF and IG algorithms are based on document frequency, and *t*-Test algorithm is based on term frequency. CTD and SCIW algorithms consider the category information, and they both have good accuracies.

Therefore, we conclude the following ones:

1. A term, which frequently occurs in a single class and does not occur in the other classes, is distinctive. Therefore, it should be given a high score.
2. A term, which rarely occurs in a single class and does not occur in the other classes, is irrelevant. Therefore, it should be given a low score.
3. A term, which frequently occurs in all classes, is irrelevant. Therefore, it should be given a low score.
4. A term, which occurs in some classes, is relatively distinctive. Therefore, it should be given a relatively high score.

From points (1) and (2), it can be seen that high-frequency terms have effects on the classification performance. From points (3) and (4), it can be seen that category information is also a very important factor which influences the classification effect. As a result, we have a conclusion that high-frequency terms and category information are both very important factors in improving the classification performance. In view of these, high-frequency terms and category information are considered synthetically when constructing feature selection function in this paper. When judging whether a word is a high-frequency term, term frequency method is used. While considering category information, we notice that ① if the probability that the feature *t*_*i*_ occurs in category *C*_*k*_ is higher than other features, *t*_*i*_ can represent *C*_*k*_ more effectively, ② if the probability that the feature *t*_*i*_ occurs in category *C*_*k*_ is higher than *t*_*i*_ occurs in other categories, *t*_*i*_ can represent *C*_*k*_ more effectively, ③ if the conditional probability that the feature *t*_*i*_ belongs to category *C*_*k*_ is higher than *t*_*i*_ belongs to other categories when the feature *t*_*i*_ occurs, *t*_*i*_ can represent *C*_*k*_ more effectively. So, the feature selection function constructed in this paper considers the interclass and intraclass relative contributions of terms to measure the category information.

Based on the above, we propose a new feature selection approach, IIRCT, in which term frequency and the interclass relative contribution and the intraclass relative contribution of terms are all considered synthetically.

### 3.2. Algorithm Implementation

In this section, we firstly introduce some symbols.

TF_*ik*_ is the term frequency of term *t*_*i*_ in category *C*_*k*_, and it is calculated according to

(8) $$\begin{array}{c} TF_{ik}=\overset{N_{k}}{\underset{j=1}{\sum }}tf_{ij}, \end{array}$$

where *N*_*k*_ is the document number in category *C*_*k*_ and tf_*ij*_ is the times that the term *t*_*i*_ appears in document *d*_*j*_.

df_*ik*_ is the document frequency of term *t*_*i*_ in category *C*_*k*_.

TF_*k*_ is the total term frequency of all terms in category *C*_*k*_, and the calculation formula is as follows:

(9) $$\begin{array}{c} TF_{k}=\overset{M_{k}}{\underset{i=1}{\sum }}TF_{ik}, \end{array}$$

where *M*_*k*_ is the term number in category *C*_*k*_.

df_*i*_ is the total document frequency of term *t*_*i*_ in all categories, and it is calculated according to

(10) $$\begin{array}{c} df_{i}=\overset{\left|C\right|}{\underset{k=1}{\sum }}df_{ik}, \end{array}$$

where |*C*| is the category number.

IIRCT algorithm measures the significance of a term from three aspects comprehensively, which are term frequency and the interclass and intraclass relative contributions of terms. Thus, we define comprehensive measurement for each term *t*_*i*_ with respect to category *C*_*k*_ as follows:

(11) $$\begin{array}{c} IIRCT\left(\;t_{i},C_{k}\;\right)=\overset{\left|C\right|}{\underset{j=1,j\neq k}{\sum }}\left(\;\frac{TF_{ik}}{TF_{k}}*\frac{df_{ik}}{df_{i}}-\frac{TF_{ij}}{TF_{j}}*\frac{df_{ij}}{df_{i}}\;\right), \end{array}$$

where |*C*| is the category number, TF_*ik*_ is the term frequency of term *t*_*i*_ in category *C*_*k*_, TF_*k*_ is the total term frequency of all terms in category *C*_*k*_, df_*ik*_ is the document frequency of term *t*_*i*_ in category *C*_*k*_, and df_*i*_ is the total document frequency of term *t*_*i*_ in all categories.

In view of the probability theory, we can regard TF_*ik*_/TF_*k*_ in (11) as the probability that the feature *t*_*i*_ occurs in category *C*_*k*_, that is, *p*(*t*_*i*_∣*C*_*k*_). df_*ik*_/df_*i*_ in (11) can be considered as the conditional probability that the feature *t*_*i*_ belongs to category *C*_*k*_ when the feature *t*_*i*_ occurs, that is, *p*(*C*_*k*_∣*t*_*i*_). TF_*ij*_/TF_*j*_ in (11) can be considered as the probability that the feature *t*_*i*_ occurs in category *C*_*j*_, that is, *p*(*t*_*i*_∣*C*_*j*_). df_*ij*_/df_*i*_ in (11) can be considered as the conditional probability that the feature *t*_*i*_ belongs to category *C*_*j*_ when the feature *t*_*i*_ occurs, that is, *p*(*C*_*j*_∣*t*_*i*_). So (11) can be further represented as follows:

(12) IIRCTti,Ck=∑j=1,j≠kCpti ∣ Ck∗pCk ∣ ti−pti ∣ Cj∗pCj ∣ ti.

Here, *p*(*t*_*i*_∣*C*_*k*_) is the probability that the feature *t*_*i*_ occurs in category *C*_*k*_, and *p*(*C*_*k*_∣*t*_*i*_) can be considered as the conditional probability that the feature *t*_*i*_ belongs to category *C*_*k*_ when the feature *t*_*i*_ occurs.

To measure the goodness of a term globally, we construct the following function:

(13) $$\begin{array}{c} IIRCT\left(\;t_{i}\;\right)=\overset{\left|C\right|}{\underset{k=1}{\sum }}p\left(\;C_{k}\;\right)*IIRCT\left(\;t_{i},C_{k}\;\right), \end{array}$$

where *p*(*C*_*k*_) = *N*_*k*_/*N* which is the probability that category *C*_*k*_ occurs in the entire training set, *N*_*k*_ is the document number in category *C*_*k*_, and *N* is the document number in collection *D*.

### 3.3. Algorithm Description

According to the above, we present a new feature selection algorithm, IIRCT, based on interclass and intraclass relative contributions of terms. Its pseudocode is as in Pseudocode 1.

## 4. Experiments Setup

### 4.1. Experimental Data

In this paper, we use two popular datasets, 20 NewsGroup and SougouCS.

The 20 NewsGroup corpus, which is collected by Ken Lang, has been widely used in text classification. This corpus contains 19997 newsgroup documents which are nearly evenly distributed among 20 discussion groups, and every group consists of 1,000 documents. All letters are converted into lowercase, and the word stemming is applied. In addition, we use the stop words list to filter words. The details of 20 NewsGroup corpus are as shown in Table 1.

The SougouCS corpus is provided by Sogou Laboratory. The documents of the corpus are from Sohu news website which has a lot of classified information. As the number of web pages in some classes is too small, we only choose 12 classes. And the detail is as shown in Table 2.

### 4.2. Document Representation

Documents are represented by vector space model [4]. That is, the content of a document is represented by a vector in the term space. It is illustrated in detail as the following. Consider *V*(*d*) = (*t*_1_, *w*_1_(*d*),…, *t*_*i*_, *w*_*i*_(*d*),…, *t*_*m*_, *w*_*m*_(*d*)), where *m* is the number of the features selected by feature selection algorithms and *w*_*i*_(*d*) is the weight of feature *t*_*i*_ in document *d*. In experiments, Term Frequency-Inverse Document Frequency (TF-IDF) [18] is used to calculate the weights of the m selected features in each document.

### 4.3. Classifier Selection

In the experiments, *k*-Nearest Neighbors (kNN) is used to classify and test documents. And it is also a case-based or instance-based categorization algorithm. At present, kNN is widely used in text classification as it is simple and has low error rate.

The principle of kNN classification algorithm is very simple and intuitive. Giving a test document whose category is unknown, the classification system will find the *k*-nearest documents by computing the similarities between documents in training data. And then, we will get the category of the test documents according to the *k*-nearest documents. The similarity measure used for the classifier is the cosine function [19].

In the paper, we set *k* = 20. And we randomly select 65% instances from each category as training data and the rest as testing data.

### 4.4. Performance Measures

We measure the effectiveness of classifiers in terms of the combination of precision (*p*) and recall (*r*) widely used in text categorization. That is, we use the well-known *F*_1_ function [20] as follows:

(14) $$\begin{array}{c} F_{1}=\frac{2*p*r}{p+r}. \end{array}$$

For multiclass text categorization, *F*_1_ is usually calculated in two ways. And they are the macroaveraged *F*_1_ (macro-*F*_1_) and the microaveraged *F*_1_ (micro-*F*_1_). Here, we only use macro-*F*_1_, as shown in

(15) $$\begin{array}{c} macro\text{-}F_{1}=\frac{\sum_{k=1}^{K}F_{1}\left(k\right)}{K}, \end{array}$$

where *F*_1_(*k*) is the *F*_1_ value of the predicted *k*th category.

## 5. Results and Discussions

### 5.1. Results and Discussions on 20 NewsGroup

**Figure 1:**
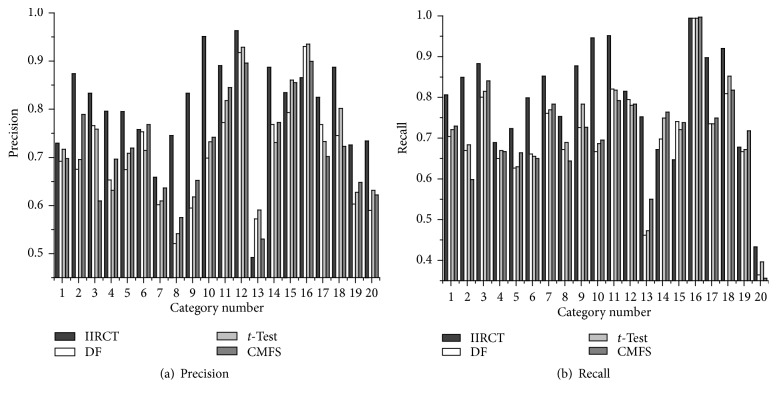
Precision and recall performance on the 20 NewsGroup corpus.

Figure 1 shows the precision and recall of IIRCT, DF, *t*-Test, and CMFS on the 20 NewsGroup corpus when 1,500 features are selected in feature space. It can be seen from Figure 1(a) that the precision of IIRCT is higher than that of DF, *t*-Test, and CMFS. And in some categories, the precision of IIRCT almost reaches up to 95%. Similarly, Figure 1(b) also indicates that the performance of IIRCT is better than that of DF, *t*-Test, and CMFS, and the recall of most categories has some improvements.

The numbers 1–20 in Figure 1 can be referred to in Table 1.

**Figure 2:**
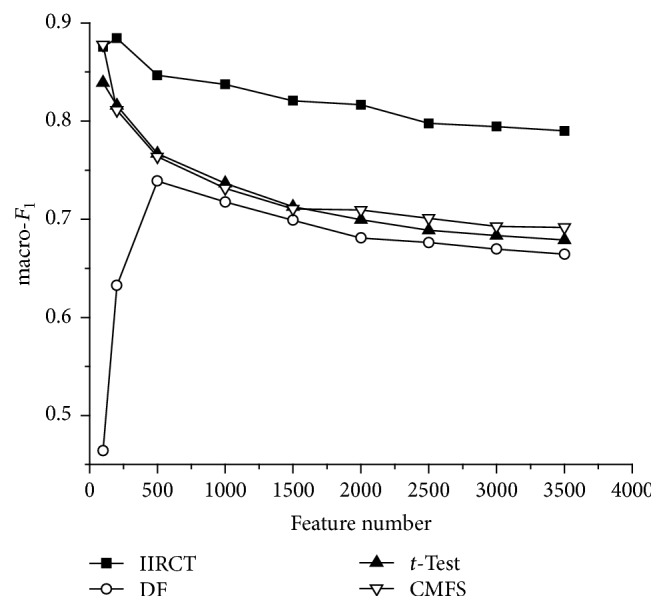
macro-*F*_1_ performance on the 20 NewsGroup corpus.

Figure 2 shows the macro-*F*_1_ performance of IIRCT, DF, *t*-Test, and CMFS on the 20 NewsGroup corpus with different feature dimensionalities. From Figure 2, we can conclude that the macro-*F*_1_ of IIRCT is close to that of CMFS when 100 features are selected. But if 200, 500, 1000, 1500, 2000, 2500, 3000, or 3500 terms are selected as features, the macro-*F*_1_ curve of IIRCT is higher than that of DF, *t*-Test, and CMFS. This means that the performance of IIRCT is better than the other three algorithms. Besides, it can be found that the value of macro-*F*_1_ decreases as the feature number increases. The reason for this is that the boundaries between categories are very clear in the 20 NewsGroup corpus. As a consequence, small amount of features can achieve good classification performance. But with the feature number increasing, many features have a negative impact on classification performance. And the classification effect gets poor.

### 5.2. Results and Discussions on SougouCS

**Figure 3:**
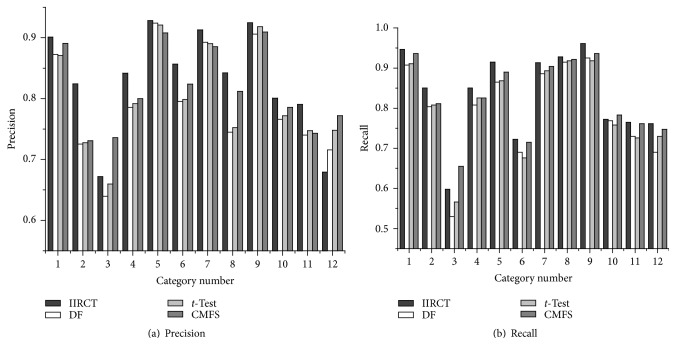
Precision and recall performance on the SougouCS corpus.

Figure 3 shows the precision and recall of IIRCT, DF, *t*-Test, and CMFS on the SougouCS corpus when 4,500 features are selected in feature space. It is clear that, in most categories, the precision and recall of IIRCT have some improvements compared to DF, *t*-Test, and CMFS. And this means that IIRCT achieves better performance than that of DF, *t*-Test, and CMFS.

The numbers 1–12 in Figure 3 can be referred to in Table 2.

**Figure 4:**
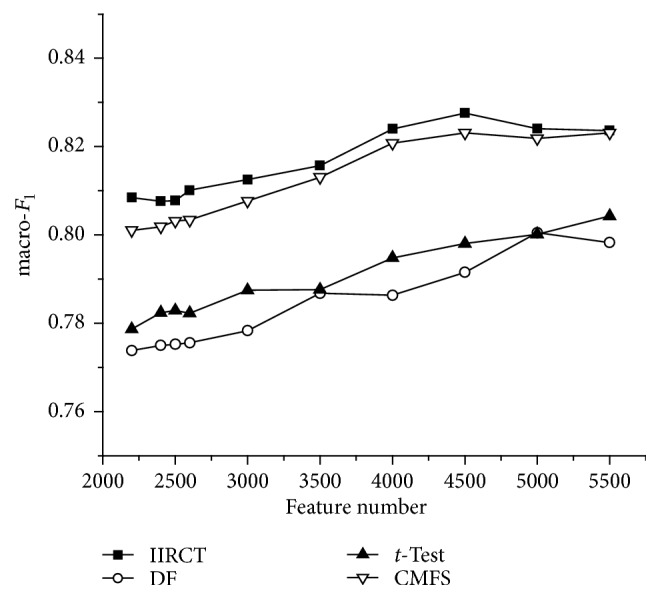
macro-*F*_1_ performance on the SougouCS corpus.

Figure 4 depicts the macro-*F*_1_ performance of the four algorithms on the SougouCS corpus. From Figure 4, we can know that the macro-*F*_1_ curve of IIRCT lies above the other three curves, which also means IIRCT has better performance than that of DF, *t*-Test, and CMFS. Besides, it can be found that the value of macro-*F*_1_ is the largest when 4500 features are selected. And when the selected feature number increases or decreases from 4500, the value of macro-*F*_1_ decreases. The reason for this is that, in the SougouCS corpus, some categories, such as fashion and entertainment, have many common words which make the boundaries between categories obscure. When small amount of features is selected, some documents cannot be classified correctly. And when the feature number increases to a certain value, these features make the boundaries between categories clear and improve the classification effect. When the feature number keeps increasing, many features have a negative impact on classification performance. And the classification effect gets poor.

## 6. Conclusions

Feature selection plays a critical role in text classification and has an immediate impact on text categorization. So we put forward a feature selection approach, IIRCT, based on interclass and intraclass relative contributions of terms in the paper. In our proposed algorithm, term frequency and the interclass and intraclass relative contributions of terms are all considered synthetically. The experimental results on 20 NewsGroup and SougouCS corpora show that IIRCT achieves better performance than DF, *t*-Test, and CMFS. Therefore, the algorithm proposed in this paper is an effective feature selection method.

## Figures and Tables

**Pseudocode 1:**
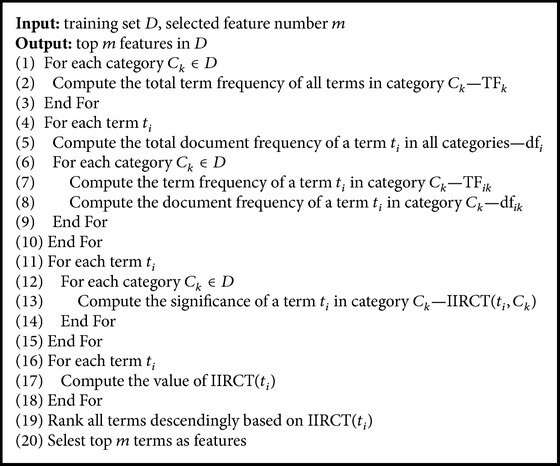


**Table 1:** 20 NewsGroup corpus.

Category number	Category name
1	alt.atheism
2	comp.graphics
3	comp.os.ms-windows.misc
4	comp.sys.ibm.pc.hardware
5	comp.sys.mac.hardware
6	comp.windows.x
7	misc.forsale
8	rec.autos
9	rec.motorcycles
10	rec.sport.baseball
11	rec.sport.hockey
12	sci.crypt
13	sci.electronics
14	sci.med
15	sci.space
16	soc.religion.christian
17	talk.politics.guns
18	talk.politics.mideast
19	talk.politics.misc
20	talk.religion.misc

**Table 2:** SougouCS corpus.

Category number	Category name
1	Car
2	Finance
3	IT
4	Health
5	Sports
6	Tourism
7	Education
8	Culture
9	Military
10	Housing
11	Entertainment
12	Fashion
